# Tracing Africa’s progress towards implementing the Non-Communicable Diseases Global action plan 2013–2020: a synthesis of WHO country profile reports

**DOI:** 10.1186/s12889-017-4199-6

**Published:** 2017-04-05

**Authors:** Gertrude Nsorma Nyaaba, Karien Stronks, Ama de-Graft Aikins, Andre Pascal Kengne, Charles Agyemang

**Affiliations:** 1 Department of Public Health, Academic Medical Centre, University of Amsterdam, Meibergdreef 9, 1105 AZ Amsterdam, The Netherlands; 2 Department of Public Health; Academic Medical Centre, University of Amsterdam, PO-box 22660, 1100 DD Amsterdam, The Netherlands; 3 Regional Institute for Population Studies, University of Ghana, P. O. Box LG 96, Legon, Ghana; 4 Non-Communicable Diseases Research Unit, South African Medical Research Council & University of Cape Town, Cape Town, South Africa; 5 Department of Public Health, Academic Medical Centre, University of Amsterdam, Meibergdreef 15, 1105 AZ Amsterdam, The Netherlands

**Keywords:** Non-communicable diseases, Africa, WHO, NCD Health Policy, Multi-sectoral NCD strategy

## Abstract

**Background:**

Half of the estimated annual 28 million non-communicable diseases (NCDs) deaths in low- and middle-income countries (LMICs) are attributed to weak health systems. Current health policy responses to NCDs are fragmented and vertical particularly in the African region. The World Health Organization (WHO) led NCDs Global action plan 2013–2020 has been recommended for reducing the NCD burden but it is unclear whether Africa is on track in its implementation. This paper synthesizes Africa’s progress towards WHO policy recommendations for reducing the NCD burden.

**Methods:**

Data from the WHO 2011, 2014 and 2015 NCD reports were used for this analysis. We synthesized results by targets descriptions in the three reports and included indicators for which we could trace progress in at least two of the three reports.

**Results:**

More than half of the African countries did not achieve the set targets for 2015 and slow progress had been made towards the 2016 targets as of December 2013. Some gains were made in implementing national public awareness programmes on diet and/or physical activity, however limited progress was made on guidelines for management of NCD and drug therapy and counselling. While all regions in Africa show waning trends in fully achieving the NCD indicators in general, the Southern African region appears to have made the least progress while the Northern African region appears to be the most progressive.

**Conclusion:**

Our findings suggest that Africa is off track in achieving the NCDs indicators by the set deadlines. To make sustained public health gains, more effort and commitment is urgently needed from governments, partners and societies to implement these recommendations in a broader strategy. While donors need to suit NCD advocacy with funding, African institutions such as The African Union (AU) and other sub-regional bodies such as West African Health Organization (WAHO) and various country offices could potentially play stronger roles in advocating for more NCD policy efforts in Africa.

**Electronic supplementary material:**

The online version of this article (doi:10.1186/s12889-017-4199-6) contains supplementary material, which is available to authorized users.

## Background

Non communicable diseases (NCDs) kill 38 million people annually worldwide with 80% of these deaths occurring and often prematurely, in low- and middle-income countries (LMIC) where more than 70% of the world’s poorest people live and access to integrated primary health care programmes and effective and equitable health care services are limited [1–4]. Exposure to known risk factors account for about two-thirds of premature NCDs deaths with an estimated half of NCDs deaths attributed to weak health systems in LMIC [1–4]. LMIC are more vulnerable to NCDs as they offer the least capacity to cope with the increasing burden due to poverty [5]. LMIC are four times less likely to have NCDs services covered by health insurance than high-income countries with their exorbitant costs further entrenching poverty as NCDs affect the poorest people in LMIC.

Eighty percent of the 54 African countries are classified by the World Bank as LMIC [6]. Africa is expected to experience the largest increase in NCD related mortality globally: about 46% of all mortality in Africa is expected to be attributed to NCDs by 2030 [7, 8]. Unless urgent action is taken, the rising NCDs burden will add great pressure to overstretched health systems and pose a major challenge to development in Africa. While low cost solutions and high impact essential NCDs interventions delivered through primary health-care approach have been shown to have population level impacts, existing literature shows that health policy responses to NCDs has been inadequate, vertical and fragmented [5, 7, 9–13].

Creating healthy public policies and reorienting health systems to address the needs of people with NCDs is crucial in NCD prevention and control [3, 14], which requires governments to play leading roles in developing and implementing comprehensive policy approaches.

The World Health Organization (WHO) in April 2011, published the WHO Global status report on NCDs 2010 which provided a description of the global burden of NCDs, their risk factors and determinants [15]. This report triggered the adoption of a political declaration at the first United Nations (UN) General Assembly high-level meeting on NCDs in 2011 [14, 15]. Consequently the NCDs country profiles was published in September 2011 to provide individual member countries NCD situational analysis [15]. This report subsequently resulted in the NCDs Global Action Plan 2013–2020 which was released in 2013 and further reinforced by the 2030 agenda [14].

In July 2014, the NCD country profiles 2014 was published to provide an update of each member state NCD status as at December 2013 so as to assess progress towards the NCDs Global Action Plan 2013–2020 and identify blockages and priority actions [8]. The report identified differences in socioeconomic development and progress in NCD prevention and control and stressed the shared benefits of comprehensive NCD responses. It formed the basis for the second high-level UN General Assembly meeting in 2014, which resulted into the 2014 Outcome Document where member state countries committed to setting national NCD targets by 2015 and implementing the WHO recommended NCD policy responses.

Building on the NCD targets of the 2011 UN Political Declaration and the 2014 Outcome Document, ten indicators made up of 18 targets were developed by the WHO in 2015 to facilitate uniform reporting and promote accountability in measuring and reporting member states’ NCD prevention and control progress [4, 14]. The ten indicators are time-bound and expected to be fully achieved by 2015 and 2016. They focus on setting of overall NCD reduction targets, strong measures to reduce tobacco consumption, harmful use of alcohol, unhealthy diets and physical inactivity and measures to strengthen treatment and care for people with NCDs [16]. They are a combination of health care measures targeting the premature NCD burden and providing the health care needs of high risk people and people living with NCDs. They will form the basis for WHO reporting at the 2017 UN General Assembly and at the 2018 high-level UN General Assembly meeting. Subsequently, an NCD country profiles report was published in September 2015 which outline individual member countries NCD progress towards the ten indicators [14]. The report shows that globally, 14 countries had not achieved any indicator, 12 of which were in Africa while 20 countries had only achieved one indicator, 15 of which were in Africa [16].

In the last few years several reviews have addressed the structural responses to NCDs in world regions such as the Americas and the Caribbean [12]. A review conducted in the Americas, for example, found significant regional progress resulting from the commitment of governments to formulating and implementing NCDs policy in the Americas [16]. Although Africa is expected to experience the largest increase in NCD related mortality globally, there are limited reviews on it's structural responses to NCDs. We therefore sort to answer the research question “what is Africa’s policy progress in implementing the WHO NCD recommendations towards achieving the NCDs Global Action Plan 2013-2020?”

## Methods

We reviewed African countries NCD data drawn from three WHO published reports; NCD country profiles 2011, NCD country profiles 2014 and the NCD country profiles 2015. In view of readability, we mentioned these as 2011, 2014 and 2015 report. WHO published the three reports containing data collected using the WHO NCD country capacity survey (CCS) conducted in 2009–2010, 2013 and 2015. The WHO CCS is completed by NCD focal points or designated colleagues within the Ministry of Health (MoH) or a national health institute/agency in each member states and submitted to WHO for review in line with the Global NCD Action Plan. WHO cross referenced the data collected with other sources including the UN Population Division World Population Prospects (population estimates), the World Bank (income group data), and the UN World Urbanization Prospects (percentage of population living in urban areas). Discrepancies in data were clarified with individual countries with supporting documents provided to WHO for validation. Prior to publication of each report; countries had the opportunity to update their profiles and endorse the results presented. The 2011 report focuses on the NCD situation of member countries [15], the 2014 report focuses on operational/functioning efforts of NCD control plans [8] while the 2015 report assesses progress against the 18 targets which make up the WHO 10 developed progress monitoring indicators [14].

Data on all 54 African countries were extracted from the NCDs country profiles 2011, 2014 and 2015 accessed from WHO website (http://www.who.int/nmh/publications/en/) into an excel sheet. We synthesized targets by their descriptions in each of the three reports and included only indicators for which we could trace progress in at least two of the three reports as shown in Additional file 1. While the 18 measures which make up the 10 monitoring indicators were our main reference, we excluded indicators and measures for which we could not trace in at least two reports. The data were then analysed using SPSS to generate descriptive statistics of achievements. Definitions for full or partial achievement varied for each indicator as detailed in Additional files 2, 3, 4 and 5. Countries were also grouped by region (East, West, North, South and Central African regions) and frequencies and percentages of achievements were generated by region.

## Results

### Characteristics

Data from 53 African countries in the 2011 report and 54 countries in the 2014 and 2015 reports were included in this analysis. South Sudan gained independence from Sudan in 2011 which accounts for the increase in number of countries reported. Equatorial Guinea was the only high income country.

### Indicator 1: Set time-bound national targets and indicators based on WHO guidance by 2015

As one of the earliest targets, 91% of African countries had established an NCD unit, branch or department within the Ministry of Health (MOH) or its equivalent in the 2011 report with only 32% of African countries having functional NCD units in the 2014 report (Table 1). In the 2015 report, only 18.5% of African countries had fully set national NCD indicators which are time-bound, based on the nine global targets, and addressed NCD mortality and key risk factors. In addition, 9.3% of African countries had partly achieved this indicator (Table 1). Guinea, Libya, Niger, Somalia, Sudan, Zimbabwe and Sao Tome and Principe are included in the none achieving countries in the 2015 report.

**Table 1:** Progress toward indicators 1–4 set to be acheived by 2015

	Ind 1			Ind 2		Ind 3		Ind 4	
Acheivement	2011	2014	2015	2011	2015	2011	2015	2014	2015
Overall Progress (2011: *N* = 53, 2014&2015 *N* = 54)									
Fully achieved	48(91%)	17(31%)	10(19%)	36(68%)	2(4%)	17(32%)	5(9%)	7(13%)	12(22%)
Partly achieved	NA	NA	5(9%)	NA	5(9%)	NA	29(54%)	NA	2(4%)
Not achieved	5(9%)	37(69%)	39(72%)	17(32%)	47(87%)	36(68%)	20(37%)	47(87%)	40(74%)
By Regions									
East African Region (2011: *N* = 17, 2014 & 2015: *N* = 18)									
Fully achieved	16 (94%)	9 (50%)	4 (22%)	12 (71%)	1 (6%)	4 (24%)	3 (17%)	3 (17%)	5 (28%)
Partly achieved	NA	NA	1 (6%)	NA	1 (6%)	NA	10 (56%)	NA	1 (6%)
Not achieved	1 (6%)	9 (50%)	13 (72%)	5 (29%)	16 (89%)	13 (76%)	5 (28%)	15 (83%)	12 (67%)
West African Region (*N* = 16)									
Fully achieved	14 (88%)	3 (19%)	2(13%)	12(75%)	1(6%)	5(31%)	1(6%)	4(25%)	3(19%)
Partly achieved	NA	NA	2(13%)	NA	2(13%)	NA	6(38%)	NA	0
Not achieved	2(13%)	13 (81%)	12(75%)	4(25%)	13(81%)	11(69%)	9(56%)	12(75%)	13(81%)
North African Region (*N* = 6)									
Fully achieved	5 (83%)	2(33%)	1(17%)	4(67%)	0	3(50%)	1(17%)	0	1(17%)
Partly achieved	NA	NA	2(33%)	NA	1(17%)	NA	4(67%)	NA	0
Not achieved	1 (17%)	4	3(50%)	2(33%)	5(83%)	3(50%)	1(17%)	6(100%)	5(83%)
South African Region (*N* = 5)									
Fully achieved	5 (100%)	3(60%)	1(20%)	4(80%)	0	2(40%)	0	0	1(20%)
Partly achieved	NA	NA	0	NA	0	NA	3(60%)	NA	0
Not achieved	0	2(40%)	4(80%)	1(20%)	5(100%)	3(60%)	2(40%)	5(100%)	4(80%)
Central African Region (*N* = 9)									
Fully achieved	8 (89%)	0	2(22%)	4(44%)	0	3(33%)	0	0	2(22%)
Partly achieved	NA	NA	0	NA	1(11%)	NA	6(67%)	NA	1(11%)
Not achieved	1(11%)	9(100%)	7(78%)	5(56%)	8(89%)	6(67%)	3(33%)	9(100%)	6(67%)

*NCD Country profiles reports 2011 and 2014 reports did not report on partial achievements (NA)

As shown in Table 1, while every country in the Southern African region had established an NCD unit, branch or department within the MOH or its equivalent in the 2011 report, only one country (South Africa) had set time-bound national NCD indicators based on the 9 global targets, address NCD mortality and the key risk factors in 2015 report. The Central African region recorded no achievements in the 2014 report but had the highest regional achievement of 22% equally achieved by the Eastern African region in the 2015 report.

### Indicator 2: Have a functioning system for generating reliable cause-specific mortality data on a routine basis by 2015

Although 68% of African countries included cause–specific NCD mortality in the national health reporting system (Table 1) in the 2011 report, only 3.7% had fully functional systems for generating reliable cause-specific mortality data on routine basis in the 2015 report. While the majority of African countries did not achieve any of these conditions, an additional 9% of African countries had partly achieved this by having met one or two of the three conditions needed. No country in the Southern African region had met at least one of the three conditions needed for achieving this indicator in the 2015 report as seen in Table 1.

### Indicator 3: Have a STEPwise approach to Surveillance (STEPS) survey or a comprehensive health examination survey every 5 years by 2015

Thirty-two percent of African countries had included NCD risk factors in their national health reporting systems in the 2011 report; only 9.3% of them had fully implemented a STEPS survey in the 2015 report (Table 1). In addition, 53.7% of the countries had partly established STEPS surveys. The Democratic Republic of Congo, Guinea, Namibia and Sudan were none achieving countries in the 2015 report. While 50% of North African region had the highest achievement of including NCD risk factors into their national health reporting systems in the 2011 report, the East African region had the highest achievement of 17% in the 2015 report. In addition, as shown in Table 1, all regions made progress in partial achievements on this indicator.

### Indicator 4: Operational multi-sectoral national strategy/action plan that integrates the major NCDs and their shared risk factors by 2015

Assessment of efforts towards this indicator included data from the 2014 and 2015 reports. While 68.5% of African countries had operational multi-sectoral national policies, strategies or action plans that integrated several NCDs and shared risk factors in the 2014 report, only 22.2% of them had fully operational national integrated NCD policies, strategies or action plans in the 2015 report (Table 1). The Democratic Republic of Congo, Guinea, Namibia, and Sudan were among the none achieving countries in the 2015 report. Twenty-five percent of the Western African region and 17.6% of the Eastern African region had operational multi-sectoral national policies, strategies or action plans that integrated several NCDs and shared risk factors in the 2014 report. Although the majority of achievements were yet to be validated by WHO at the time of the 2015 report, the Eastern African region had the highest achievements of 28% with the remaining regions recording at least one country each as shown in Table 1.

### Indicator 5: Implemented the following four demand-reduction measures of the WHO FCTC at the highest level of achievement by 2016:

Four measures are proposed for the FCTC for reducing/regulating tobacco use, only two met our selection criteria (Additional file 1).

- **❖** *Reduce affordability of tobacco products by increasing tobacco excise taxes*
  In the 2011 and 2014 reports, approximately 4 out of 10 of African countries had operationally integrated or tobacco-specific policies/programs/action plans with only Tunisia having fully reduced the affordability of tobacco products by increasing tobacco excise taxes in the 2015 report. In addition, only 13% of African countries had partly reduce the affordability of tobacco products by setting tobacco excise taxes of at least 50% but less than 70% of the retail price (Table 2). The Southern African region recorded the least achievements in all three years.
- **❖** *Warn people of the dangers of tobacco and tobacco smoke through effective health warnings and mass media campaigns*
  In the 2011 report, 64% of Africa had allocated funding for NCD prevention and health promotion activities but only 13% of these countries had fully warned people of the dangers of tobacco and tobacco smoke through effective health warnings and mass media campaigns in the 2015 report. Thirty-five percent of African countries had partly warned people of the dangers of tobacco and tobacco smoke through effective health warnings and mass media campaigns in the 2015 report. While Eritrea, Gambia, Liberia, Senegal, Togo, Tunisia and Seychelles had fully achieved this indicator in 2015, only Tunisia achieved this indicator in all three reports. In the 2015 report, the Western African region took the lead with 25% of its countries achieving this sub-indicator fully while the Southern African region recorded no full achievements in the 2015 report as shown in Table 2. Sixty percent of the Southern African region however made partial achievements for this indicator.

**Table 2:** Progress towrads indicators 5 and 6 set to be achieved by 2016

	Ind 5							Ind 6				
Acheivement	2011	2014	a.2015	b.2015	c.2011	c.2015	d.2015	2011	2014	a.2015	b.2015	c.2015
Overall Progress (2011: *N* = 53, 2014&2015 *N* = 54)												
Fully achieved	20(38%)	20(37%)	1(2%)	7(13%)	34(64%)	7(13%)	11(20%)	12(23%)	11(20%)	10(19%)	8(15%)	13(24%)
Partly achieved	NA	NA	7(13%)	18(33%)	NA	19(35%)	22(41%)	NA	NA	39(72%)	17(31%)	31(72%)
Not achieved	33(63%)	34(64%)	46(85%)	29(54%)	19(36%)	28(52%)	21(39%)	41(77%)	43(80%)	5(9%)	29(54%)	5(9%)
By Regions												
East African Region (2011: *N* = 17, 2014 & 2015: *N* = 18)												
Fully achieved	6(35%)	4(22%)	0	1(6%)	9(53%)	2(11%)	3(17%)	4(24%)	4(22%)	2(11%)	1(6%)	3(17%)
Partly achieved	NA	NA	1(6%)	7(39%)	NA	7(39%)	8(44%)	NA	NA	15(83%)	7(39%)	12(67%)
Not achieved	11(65%)	14(78%)	17(94%)	10(56%)	8(47%)	9(50%)	7(39%)	13(76%)	14(78%)	1(6%)	10(56%)	3(17%)
West African Region (*N* = 16)												
Fully achieved	8(50%)	7(44%)	0	4(25%)	10(63%)	4(25%)	5(31%)	4(25%)	4(25%)	5(31%)	3(19%)	5(31%)
Partly achieved	NA	NA	4(25%)	5(31%)	NA	3(19%)	5(31%)	NA	NA	9(56%)	6(38%)	7(44%)
Not achieved	8(50%)	9(56%)	12(75%)	7(44%)	6(38%)	9(56%)	6(38%)	12(75%)	12(75%)	2(13%)	7(44%)	4(25%)
North African Region (*N* = 6)												
Fully achieved	3(50%)	5(83%)	1(17%)	0	5(83%)	1(17%)	0	2(33%)	1(17%)	0	1(17%)	2(33%)
Partly achieved	NA	NA	0	4(67%)	NA	3(50%)	3(50%)	NA	NA	6(100%)	1(17%)	4(67%)
Not achieved	3(50%)	1(17%)	5(83%)	2(33%)	1(17%)	2(33%)	3(50%)	4(67%)	5(83%)	0	4(67%)	0
South African Region (*N* = 5)												
Fully achieved	1(20%)	1(20%)	0	2(40%)	4(80%)	0	2(40%)	1(20%)	0	0	0	0
Partly achieved	NA	NA	0	0	NA	3(60%)	2(40%)	NA	NA	4(80%)	0	3(60%)
Not achieved	4(80%)	4(80%)	5(100%)	3(60%)	1(20%)	2(40%)	1(20%)	4(80%)	5(100%)	1(20%)	5(100%)	2(40%)
Central African Region (*N* = 9)												
Fully achieved	2(22%)	3(38%)	0	0	6(67%)	0	1(11%)	1(11%)	2(22%)	3(33%)	3(33%)	3(33%)
Partly achieved	NA	NA	2(22%)	2(22%)	NA	3(33%)	4(44%)	NA	NA	5(63%)	3(33%)	5(63%)
Not achieved	7(78%)	5(63%)	7(78%)	7(78%)	3(33%)	6(67%)	4(44%)	8(89%)	7(78%)	1(11%)	3(33%)	1(11%)

*NCD Country profiles reports 2011 and 2014 reports did not report on partial achievements (NA)

### Indicator 6: Implemented, as appropriate according to national circumstances, three measures to reduce the harmful use of alcohol as per the WHO Global Strategy to Reduce the Harmful Use of Alcohol by 2016

Three measures are proposed to reduce the harmful use of alcohol none of which met our selection criteria. However, both the 2011 and 2014 reports assessed reducing harmful use of alcohol indicator as one indicator and thus met our selection criteria (Additional file 1).

Twenty-three percent of African countries had an operationally integrated or topic-specific policy/programme/action plan for alcohol in the 2011 report. In the 2014 report, 20% of African countries had operational policies, strategies or action plans to reduce the harmful use of alcohol. Regionally, while the Northern Africa region recorded 33.3% as the highest achievements in the 2011 report, the West African region recorded the highest achievement in the 2014 report with 25% of its countries having operationally integrated or alcohol -specific policies/programs/action plans. The Central African region recorded the lowest achievement with one country in the 2011 report and the Southern African region recorded no achievements in the 2014 report as shown in Table 2.

### Indicator 7: Implemented four measures to reduce unhealthy diets by 2016

We assessed the measure used in the 2011 and 2014 reports as the three specific measures assessed in the 2015 report did not meet our selection criteria as shown in Additional file 1.

In the 2011 report, 32% of African countries had operationally integrated topic-specific policy/programme/action plan for unhealthy dieting, overweight and/or obesity but in the 2014 report, this was reduced to 28% (Table 3). The Northern African region had the highest achievements of 50% while only one country in the Southern African region had achieved this in the 2011 report. In the 2014 report, the Western African region had the highest achievement of 38% while the Southern African region had no achievements (Table 3).

**Table 3:** Progress towards indicators 7–9 set to be achieved by 2016

	Ind 7						Ind 8			Ind 9	
Acheivement	2011	2014	a.2015	b.2015	c.2015	d.2015	2011	2014	2015	2014	2015
Overall Progress (2011: *N* = 53, 2014&2015 *N* = 54)											
Fully achieved	17(32%)	15(28%)	1(2%)	1(2%)	1(2%)	1(2%)	15(28%)	13(24%)	16(30%)	9(17%)	4(7%)
Partly achieved	NA	NA	0	0	0	0	NA	NA	0	NA	6(11%)
Not achieved	36(68%)	39(72%)	53(98%)	53(98%)	53(98%)	53(98%)	38(72%)	41(76%)	38(70%)	45(83%)	44(81%)
By Regions											
East African Region (2011: *N* = 17, 2014 & 2015: *N* = 18)											
Fully achieved	5(29%)	4(22%)	1(6%)	0	0	1(6%)	4(22%)	4(22%)	7(39%)	4(22%)	0
Partly achieved	NA	NA	0	0	0	0	NA	NA	0	NA	1(6%)
Not achieved	12(71%)	14(78%)	17(94%)	18(100%)	18(100%)	17(94%)	13(76%)	14(78%)	11(65%)	14(78%)	16(94%)
West African Region (*N* = 16)											
Fully achieved	6(38%)	5(31%)	0	0	0	0	6(38%)	6(38%)	2(13%)	2(13%)	1(6%)
Partly achieved	NA	NA	0	0	0	0	NA	NA	0	NA	3(19%)
Not achieved	10(63%)	11(69%)	16(100%)	16(100%)	16(100%)	16(100%)	10(63%)	10(63%)	14(88%)	14(88%)	12(75%)
North African Region (*N* = 6)											
Fully achieved	3(50%)	2(33%)	0	0	0	1(17%)	3(50%)	2(33%)	2(33%)	2(33%)	0
Partly achieved	NA	NA	0	0	0	0	NA	NA	0	NA	1(17%)
Not achieved	3(50%)	4(67%)	6(100%)	6(100%)	6(100%)	5(83%)	3(50%)	4(67%)	4(67%)	4(67%)	5(83%)
South African Region (*N* = 5)											
Fully achieved	1(20%)	0	0	0	0	0	1(20%)	0	1(20%)	0	0
Partly achieved	NA	NA	0	0	0	0	NA	NA	0	NA	0
Not achieved	4(80%)	5(100%)	5(100%)	5(100%)	5(100%)	5(100%)	4(80%)	5(100%)	4(80%)	5(100%)	5(100%)
Central African Region (*N* = 9)											
Fully achieved	2(22%)	2(22%)	0	1(11%)	0	1(11%)	1(11%)	3(33%)	4(44%)	1(11%)	1(11%)
Partly achieved	NA	NA	0	0	0	0	NA	NA	0	NA	1(11%)
Not achieved	7(78%)	7(78%)	9(100%)	8(89%)	9(100%)	8(89%)	8(89%)	6(67%)	5(63%)	8(89%)	6(67%)

*NCD Country profiles reports 2011 and 2014 reports did not report on partial achievements (NA)

### Indicator 8: Implemented at least one recent national public awareness programme on diet and/or physical activity by 2016

Twenty-eight percent of African counties had integrated physical activity -specific policy/programme/action plans in the 2011 report, with this percentage lowering to 24% in the 2014 report. In the 2015 report, 30% of African countries had fully implemented at least one recent national public awareness programme on diet and/or physical activity (Table 3). From a regional perspective, Northern African region had the highest achievements while the Central and Southern African regions recorded the lowest achievements in the 2011 report. In the 2014 report, while the Southern African region did not record any achievements, the Eastern African region made the highest achievement as shown in the Table 3. In the 2015 report, 39% of the Eastern African region had fully implemented at least one recent national public awareness program on diet and/or physical activity with the Southern African region recording only one country fully achieving this indicator.

### Indicator 9: Has evidence-based national guidelines/protocols/standards for the management of major NCDs through a primary care approach, recognized/approved by government or competent authorities by 2016

Although, 17% of African countries had evidence-based national guidelines/protocols/standards for the management of major NCDs through a primary care approach in the 2014 report, the corresponding percentage was only 4% in the 2015 report. Although an additional 4% had fully achieved this indicator, this achievement was yet to be validated by WHO while 11% of African countries had achieved this indicator partially (Table 3). In the 2014 report, while the Southern African region recorded no achievements for this indicator, 34% of the Northern African region had NCD evidence-based national guidelines/protocols/standards in the 2015 report. Only the Western and Central African regions recorded one country each as having fully achieved the indicator in the 2015 report (Table 3).

## Discussion

African countries are off track in implementing their NCD commitments set to be achieved by 2015 and 2016. While Africa and indeed, its regions, recorded high achievements in the baseline year, lower achievements were recorded in subsequent reports. The highest achievements were recorded in implementing recent national public awareness programme on diet and/or physical activity set to be fully achieved by 2016 and the lowest achievements were recorded in drug therapy and evidence-based national guidelines/protocols/standards for the management of major NCDs through a primary care approach. Many countries recorded partial achievements in nearly all indicators. Despite general waning achievements for all African regions, the Northern African region appears to record more full achievements while the Southern African region appears to record the lowest progress in the indicators. These deteriorating achievements could perhaps reflect improved and more rigorous validation of achievements by WHO. It could also be argued that the ongoing political crisis in some countries such as Democratic Republic of Congo, Somalia and Sudan has contributed to effectively hindering the ability of many African governments to design and implement the needed NCD policy recommendations and resulted in the low progress achievements recorded in these countries. In addition, the low achievements recorded by the Southern African region maybe due to the focus on the heavy burden of communicable diseases such as HIV and tuberculosis and related infectious disease. Studies have suggested that despite the co-existence of communicable and NCDs, the explosive evolution of HIV/AIDS in the region redirected health resources and priorities from the progressing transition in NCDs to communicable diseases [17, 18].

More than two-thirds of African countries lack functional systems for generating cause-specific mortality data routinely which hinders the accurate estimates of disease specific burden needed for effective and appropriate health programming and delivery. Indeed, the limitations of African countries in sufficiently generating quality cause-specific mortality data have been documented. Mathers et al. reported less than 10% coverage of death registration in the African Region in their study on assessing the status of global data on death registration and its quality [19]. Another study found significant errors that affect quality of cause of death certification in South Africa and stressed the need for mortality data quality to be improved [20]. In spite of the fact that over half of African countries had conducted surveys on at least three risk factors in the last five years, accurate estimates on the NCD burden in countries remains an informed challenge for policy formulation and health service planning. Inadequate government led regulations and commitment, partnerships with key organizations, inadequate funding and competing priorities from communicable diseases could be key reasons for the poor progress. Inadequate funding and staffing for NCDs units and the lack of quality health data have been identified by several studies as leading to unproductive inter-sectoral collaboration and national level strategies needed for effective NCD control [11, 21]. To reduce costs while effectively generating evidence for policy formulation and implementation, integrating the WHO STEPs into the well-established systems for communicable diseases maybe a viable alternative. The WHO STEPs is considered a simple, standardised method for collecting, analysing and disseminating data useful for monitoring on a regular and continuing basis (http://www.who.int/chp/steps/en/). A cue should be taken from Rwanda which has made comprehensive efforts in integrating disease-specific programs such as cervical cancer, diabetes and mental health while strengthening the health system to address other conditions [10].

Although some achievements were made in having national public awareness programs, several studies have shown that these may not have significant impacts on the population or influence behaviour [22–25]. It has been suggested that such public awareness programs should be integrated into broader strategies with collaboration between all developmental stakeholders in order for such programs to achieve population level impact [22, 23, 25, 26].

Several studies have shown the increasing prevalence of smoking in Africa and the WHO FCTC identifies tax increases and health warnings as key indicators for tobacco control [27–30]. Tobacco tax increases raises government revenue and results in double decreases in tobacco consumption while health warnings/media campaigns were found to contribute to reducing tobacco use in high-income countries once integrated into tobacco control programs [31, 32]. Malawi and Somalia are not parties or signatories to the FCTC yet, but they registered partial achievements for health warnings on the dangers of tobacco in 2015. South Sudan is not signatory to the FCTC yet while Zimbabwe became a party and/or signatory to the FCTC in 2014. Many countries had partly made progress in achieving the health warnings on tobacco sub-indicator but given the limited NCD capacity in many African countries, the need for effective policies and interventions to reduce tobacco use cannot be underestimated. Active engagement and alliance between various sectors engaged in providing local evidence to support advocacy and policy, partnerships to provide public pressure for the institutional and implementation of tobacco policies are needed to ensure policy progress. These, including an in-depth understanding of the power relations and relevant structures for tobacco control policies, determination and a willingness to present a strong integrated voice have contributed to the successes of tobacco control in the Arab world [33].

To reduce the harmful use of alcohol, all member countries are encouraged to implement three key measures outlined in the WHO Global Strategy to Reduce the Harmful Use of Alcohol. While some gains were made on this indicator by 2015, more needs to be done to fully achieve these sub indicators by 2016. For instance, by December 2013, only 10 African countries had fully implemented regulations over commercial and public availability of alcohol; eight countries had fully implemented comprehensive restrictions or bans on alcohol advertising and promotions and 13 countries had fully implemented pricing policies such as tax increases on alcoholic beverages. A recent study reviewed the alcohol policies of Lesotho, Malawi, Uganda and Botswana and suggested that the progress in alcohol control in most of sub-Saharan Africa has been strongly influenced by the alcohol industry which initiated and facilitated the design and the development of most national policy documents on alcohol regulation [34]. In addition, much of the alcohol control policies have focused mainly on commercially produced alcohol to the neglect of the more common locally produced homemade alcoholic brews. Alcohol control policies often fail to incorporate regulation for these locally homemade alcoholic brews because of the difficulty in estimating alcohol content in these brews which are varied and cultural dependent. Made from local produce such as palm sap, grains or vegetables, these homemade alcoholic brews are estimated to be the highest consumed in Africa not only because they are estimated to be cheaper [35, 36] but also are used in many cultural practices. Exact estimates remain difficult due to country, regional and ethnic variations across the continent [35, 36] hindering accurate assessment of alcohol as an NCD risk factor. For effective alcohol policies that serve public health interests, a good understanding of the policy development processes, the power relations, estimates and inclusion of the traditional alcoholic home brews and an awareness of the contribution of alcohol to NCDs and hence poverty must be aligned with the interests and commitment of governments and other key stakeholders. Given that many countries had made progress in partly achieving the alcohol related sub-indicators, governments need to intensify interests and commitment to fully formulating and implementing them to make key progress on a major risk factor for NCDs.

Little progress has been made in dieting, overweight and obesity. It is probable that data on dieting and physical inactivity needed for the assessment of risks are inadequate, which impedes effective policy formulation, planning and implementation. Lachat et al.’s systematic policy review on diet and physical activity for the prevention of NCDs in LMIC reported similar findings on the low coverage of policies in Africa that were explicitly directed to at least one of the risk factors [37]. Key lessons could be learnt from countries such as Argentina, the USA, Canada, Brazil, Chile and Mexico which have succeeded in promoting salt reduction in packaged foods and bread [4].

With regards to care for people living with NCDs and targeting high risk persons such as the aged, little progress was made. Countries are required to establish guidelines for the management of major NCDs integrated into a primary care approach and drug therapy and counselling particularly for high risk persons. While no African country provides drug therapy and/or counselling for high risk persons, only Congo and Senegal had integrating the management of major NCDs into primary care. To ensure effective and sustained development and NCD programing, adequate budgets; strong national health-sector plans and policies, effective supply management, inter-sectoral coordination, access and affordability of essential drugs which are predominantly inadequate in many LMICs are needed [38]. Although some countries might have developed guidelines for the management of NCDs, integrating these guidelines into primary care may be difficult to achieve in the short term. This is due largely to the fact that the primary care approach in Africa faces many challenges including shortages in human and financial resource capacity [39]. The situation is further compounded by the political realities and cultural issues, which have been identified as further widening breaches in formulation, and implementation of NCD policies [39]. Given the intricately complex nature of NCDs, intensive advocacy is needed to facilitate government-led commitment to fully implement the polices for which many countries recorded partial achievements while ensuring that health systems are reoriented, resourced and capacities built to equip them to achieve effective NCD programing while still catering for the burden of communicable diseases on the continent. Integration into all sectors is needed to influence behaviour change and achieve population wide public health impact.

Policy responses in Africa have been slow with the biggest challenges perhaps in the reorienting of already overstretched and overburdened health systems grappling with service delivery for a high burden of communicable diseases to handle the increasing burden of NCDs. Several studies have shown the human resource crisis in health systems, reorienting service delivery towards chronic disease care, inadequate supply of equipment and medicines, low motivation and inadequate supervision as key constraints for chronic disease care [38–41]. Although, there has been some advocacy for integrating chronic disease into PHC, the challenges that these health systems face in implementing the PHC has been well documented [9, 38, 40, 41]. Effectively managing NCDs through a PHC approach requires a health system equipped with essential resources and medicines adequately trained and empowered human resources and efficient supervision of primary care facilities. While individual countries need to adapt and implement these policy recommendations in a manner appropriate for them, regional cooperation and sub-regional platforms for exchange of experiences and embracing NCD solutions and policies have been shown to contribute to effective NCD policy formation [12]. The Pan American Health Organization (PAHO) has been instrumental in supporting country-driven NCD planning and implementing while creating avenues for the Americas to share and build on each other’s experiences in their NCD efforts [12]. This has resulted in substantial achievements in implementing NCD programs with some states exceeding the set indicators of the chronic disease strategic plan [42].

The WHO Regional office for Africa (WHO-Afro) has played a key role in facilitating regional meetings and dialogues for addressing the increasing NCDs burden with significant successes in tobacco control [43]. The African Union (AU) and other sub-regional bodies such as West African Health Organisation (WAHO) and various country offices could potentially play stronger roles in advocating for more NCD policy efforts and creating the needed environment for the sharing of experiences between member countries as exemplified by PAHO. With shared policy formulations and integration, governments may become more committed to implementing the WHO recommendations for NCD prevention, control and management.

Over the last two decades, while there has been increasing international advocacy for tackling the increasing NCD burden globally and in particular in sub-Saharan Africa, this has not been equally matched with funding or financial support. While the NIH, the GlaxoSmithKline though the GSK Africa NCD Open Lab, the Grand challenge and a few other donors have tried to suit advocacy with funding support, a major shift in funding support is yet to be seen towards NCD control. An assessment of development assistance for health from 1990 to 2007 in LMIC found that funding grew from $5·6 billion in 1990 to $21·8 billion in 2007 with the bulk of global funding been directed at tackling communicable diseases such as HIV/AIDS, tuberculosis and malaria and NCDs receiving only a 3% share [44–47]. In addition, with African governments spending only 9.6% proportion of their total expenditure on health, funding for NCDs is hindered by their inability to invest more and thus facilitating catastrophic costs of people [48]. Given that donor funding most often drives health priorities in many African countries [47], inadequate donor interest and funding for NCDs could be a contributory factor for the poor progress achievements. The need for stronger advocacy matched by African governments and donor funding, accountability and transparency remains more salient if the NCD policy recommendations are to be effectively and efficient implemented particularly in sub-Saharan Africa.

### Limitations

As a synthesis of existing data derived from the WHO NCD country profile reports, this paper is constrained by the limitations of those reports and their data sources. For example, not all the monitoring sub indicators for progress could be traced from the 2011 and 2014 reports as some indicators and sub-indicators were not individually analysed in the two preceding reports. In addition, mortality and risk factor data for the NCD country profiles were estimated from World Health Statistics 2011 and 2014 reports and adjusted using standardized categories for greater comparability. The World Health Statistics 2011 and 2014 reports indicate that, for countries with weak statistical and health information systems where the quality is inadequate, the estimates were subject to considerable uncertainty. Again, WHO NCD CCS was based on self-report data which could bias the study findings. A possible way to avert this would be to have individual WHO in-country representatives complete the country capacity survey together with the NCD country representative from the health sector. Furthermore, documentation was not made available to WHO for validation and in some cases, countries reported “don’t know” or 'no data' for some of the indicators which may contribute to an underestimation of progress towards implementation for some indicators.

Furthermore, the findings of this study are based on a quantitative assessment of WHO data. Further qualitative research is needed to adequately explore the reasons for the poor progress in depth to facilitate further policy developments. Despite these limitations, this synthesis provides a critical overview of Africa’s policy responses to the increasing NCD situation which could propel African countries to urgently implement their 2011 and 2014 commitments to implement WHO NCD policy recommendations.

## Conclusion

Africa is off track in achieving the WHO recommendations for tackling NCDs set to be achieved by the 2015 and 2016 deadlines. Despite an increasing trend in NCD related morbidity and mortality, implementation of the WHO policy recommendations remains slow. It appears that despite initial achievements by African countries towards fulfilling the commitments in the 2011 UN Political Declaration and the 2014 Outcome, Africa’s commitment towards implementing the NCD policy responses has waned. These findings show Africa’s poor preparedness to handle the increasing NCD burden and although we speculate on possible reasons, further research is needed to adequately explore these reasons in depth, in order to facilitate further policy development. African institutions such as The African Union (AU) and other sub-regional bodies such as West African Health Organisation (WAHO) and various country offices could potentially play stronger roles in advocating for more NCD policy efforts in Africa.

## Additional files

Additional file 1: Selection criteria. Description of data: Selection criteria for indicators analysed. (PDF 384 kb)

Additional file 2: WHO definitions of indicators and criteria. Description of data: WHO definitions of indicators and criteria 1–4. (PDF 292 kb)

Additional file 3: WHO definitions of indicators and criteria. Description of data: WHO definitions of indicators and criteria_ 5. (PDF 212 kb)

Additional file 4: WHO definitions of indicators and criteria. Description of data: WHO definitions of indicators and criteria_6–7. (PDF 284 kb)

Additional file 5: WHO definitions of indicators and criteria. Description of data: WHO definitions of indicators and criteria_7c-10. (PDF 287 kb)

## Abbreviations

AIDS: Acquired immune deficiency syndrome
AU: African union
CCS: Country capacity survey
FCTC: Framework convention on tobacco control
GSK: GlaxoSmithKline
HIV: Human immunodeficiency virus
LMICs: Low- and middle-income countries
MoH: Ministry of Health
NCDs: Non communicable diseases
NIH: National Institute for Health
PAHO: Pan American Health Organization
PHC: Primary Health Care
SPSS: Statistical package for the social science
STEPS: STEPwise approach to Surveillance
T2D: Type 2 diabetes
UN: United Nations
WAHO: West African Health Organisation
WHO: World Health Organization
WHO-Afro: WHO Regional office for Africa
