# Supplementary material for: Tracing Africa’s progress towards implementing the Non-Communicable Diseases Global action plan 2013–2020: a synthesis of WHO country profile reports
Source: BMC Public Health. 2017 Apr 5;17:297. doi: 10.1186/s12889-017-4199-6 (PMC5382491; doi:10.1186/s12889-017-4199-6)
Supplement: Supplementary file 1 — Selection criteria. Description of data: Selection criteria for indicators analysed. (PDF 384 kb) [file 12889_2017_4199_MOESM1_ESM.pdf]

	Additional file 1: INDICATORS AND INCLUSION CRITERIA				
No	Indicators	Report year assessed			Selection criteria
		2011	*2014*	*2015*	2 ≥
1.	Set time-bound national targets and indicators based on WHO guidance by 2015	√	√	√	Included
2.	Has a functioning system for generating reliable cause-specific mortality data on a routine basis by 2015	√	x	√	Included
3.	Has a STEPS survey or a comprehensive health examination survey every 5 years	√	x	√	Included
4.	Has an operational multisectoral national strategy/action plan that integrates the major NCDs and their shared risk factors by 2015	x	√	√	Included
5.	Implemented the demand-reduction measures of the WHO FCTC at the highest level of achievement by 2015				Included
a.	Reduce affordability of tobacco products by increasing tobacco excise taxes	√	√	√	Included
b.	Create by law completely smoke-free environments in all indoor workplaces, public places and public transport	x	x	√	Excluded
c.	Warn people of the dangers of tobacco and tobacco smoke through effective health warnings and mass media campaigns	√	x	√	Included
d	Ban all forms of tobacco advertising, promotion and sponsorship	x	x	√	Excluded
6.	Implemented, as appropriate according to national circumstances, measures to reduce the harmful use of alcohol as per the WHO global Strategy to Reduce the Harmful Use of Alcohol	√	√	x	Included
a.	Regulations over commercial and public availability of alcohol	x	x	√	Excluded
b.	Comprehensive restrictions or bans on alcohol advertising and promotions	x	x	√	Excluded
c.	Pricing policies such as excise tax increases on alcoholic beverages	x	x	√	Excluded
7.	Implemented the measures to reduce unhealthy diets	√	√	x	Included
a.	Adopted national policies to reduce population salt/sodium consumption	x	x	√	Excluded
b.	Adopted national policies that limit saturated fatty acids and virtually eliminate industrially produced trans fatty acids in the food supply	x	x	√	Excluded
c.	WHO set of recommendations on marketing of foods and non-alcoholic beverages to children	x	x	√	Excluded
d.	Legislation /regulations fully implementing the International Code of Marketing of Breast-milk Substitutes	x	x	√	Excluded
8.	Implemented at least one recent national public awareness programme on diet and/or physical activity	√	√	√	Included
9.	Has evidence-based national guidelines/protocols/standards for the management of major NCDs through a primary care approach, recognized/approved by government or competent authorities	x	√	√	Included
10.	Has provision of drug therapy, including glycaemic control, and counselling for eligible persons at high risk to prevent heart attacks and strokes, with emphasis on the primary care level	x	x	√	Excluded
