# Supplementary material for: Tracing Africa’s progress towards implementing the Non-Communicable Diseases Global action plan 2013–2020: a synthesis of WHO country profile reports
Source: BMC Public Health. 2017 Apr 5;17:297. doi: 10.1186/s12889-017-4199-6 (PMC5382491; doi:10.1186/s12889-017-4199-6)
Supplement: Supplementary file 2 — WHO definitions of indicators and criteria. Description of data: WHO definitions of indicators and criteria 1–4. (PDF 292 kb) [file 12889_2017_4199_MOESM2_ESM.pdf]

Additional file 2: WHO definition of indicators and criteria				
Indicator	Definition	Achievement Criteria		
		Fully achieved	Partly achieved	Links to WHO tools
1.	Country has set national targets and indicators. The NC related targets and indicators should be time-bound and based on the 9 voluntary global targets and 25 indicators from the WHO Global Monitoring Framework.	A country has a set of national NCD indicators and has time-bound national targets for these indicators. Targets must be time-bound, based on the 9 global targets, and need to address chronic disease mortality, as well as key risk factors in the country and/or health systems.	A country has a set of national NCD indicators and time-bound national targets for these indicators but the targets do not cover two of the three areas addressed in the 9 global targets including mortality) or they are not time-bound.	http://www.who.int/chp/ncd_capacity/en/
2.	Country has a vital registration system that captures deaths and the causes of death routinely. The International Form of Medical Certificate of the Cause of Death is completed by certifiers. The International Classification of Diseases (ICD) is used to code the causes of death. The data compiled are made available to policy-makers and researchers.	Data are considered to generate reliable cause-specific mortality data on a routine basis if: • Data from the five most recent reporting years are, on average, at least 70% usable.<br>• At least five years of cause-of-death data have been reported to the WHO.<br>• The most recent year of data reported to the WHO is no more than five years old.	Not defined	http://www.who.int/healthinfo/tool_cod_2010.pdf
3.	Country has completed a STEPS survey or another risk factor survey which includes physical measurements and biochemical assessments covering the key behavioural and metabolic risk factors for NCDs. Country must indicate that survey frequency is at least every 5 years.	A country has had surveys of risk factors (may be a single RF or multiple) conducted for all of the following: Harmful alcohol use (optional for Member States according to national circumstances), Physical inactivity, Tobacco use, Raised blood glucose/diabetes, Raised blood pressure/hypertension, Overweight and obesity, and Salt /Sodium intake. Additionally, for each risk factor, the country must indicate that the last survey was conducted in the past 5 years (i.e. 2010 or later for the 2015 CCS survey responses) and must respond "Every 1 to 2 years" or "Every 3 to 5 years" to the sub-question "How often is the survey conducted?"	Country responded that at least 3, but not all, of the above risk factors are covered, or the surveys were conducted more than 5 years ago but less than 10 years ago.	http://www.who.int/chp/ncd_capacity/en/
4.	Country has a multisectoral, national integrated NCD and risk factor policy/strategy/action plan that addresses the 4 main NCDs (cardiovascular disease and/or diabetes, and/or cancer, and/or respiratory disease) and their main risk factors (tobacco use, unhealthy diet, physical inactivity, harmful use of alcohol). "Multisectoral" refers to engagement with one or more government sectors outside of health. "Operational" refers to a policy, strategy or action plan which is being used and implemented in the country, and has resources and funding available to implement it.	A country has a national NCD policy, strategy or action plan which integrates several NCDs and their risk factors which is multisectoral and operational. It must address the 4 main risk factors; Harmful use of alcohol (optional for Member States according to national circumstances); Unhealthy diet; Physical inactivity; tobacco. It must also combine early detection, treatment and care for: Cancer; Cardiovascular diseases; chronic respiratory diseases and Diabetes.	Country has a national NCD policy, strategy or action plan which integrates several NCDs and their risk factors, is multisectoral and operational. Address at least two of the 4 main risk factors and at least two of the 4 main NCDs.	http://www.who.int/chp/ncd_capacity/en/
Source : WHO Country Profiles 2015				
