# Supplementary material for: Tracing Africa’s progress towards implementing the Non-Communicable Diseases Global action plan 2013–2020: a synthesis of WHO country profile reports
Source: BMC Public Health. 2017 Apr 5;17:297. doi: 10.1186/s12889-017-4199-6 (PMC5382491; doi:10.1186/s12889-017-4199-6)
Supplement: Supplementary file 3 — WHO definitions of indicators and criteria. Description of data: WHO definitions of indicators and criteria_ 5. (PDF 212 kb) [file 12889_2017_4199_MOESM3_ESM.pdf]

Additional file 3: WHO definition of indicators and criteria				
**Indicator**	**Definition**	**Achievement Criteria**		**Links to WHO tools**
		**Fully achieved**	**Partly achieved**	**Links to WHO tools**
5.a.	Country has tobacco excise tax set at a level that accounts for at least 70% of the retail prices of tobacco products.	Country has set an excise tax at least 70% of the retail price.	Country has set an excise tax at least 50% but less than 70% of the retail price.	http://www.who.int/tobacco/global_report/ http://www.who.int/tobacco/global_report/2013/technical_note_i.pdf
5. b.	Country has all public places completely smoke-free (or at least 90% of the population covered by complete subnational smoke-free legislation). "Completely" means that smoking is not permitted, with any exemptions allowed, except in residences and indoor places that serve as equivalents to long-term residential facilities, such as prisons and long-term health and social care facilities such as psychiatric units and nursing homes. Ventilation and any form of designated smoking rooms and/or areas do not protect from the harms of second-hand tobacco smoke, and the only laws that provide protection are those that result in the complete absence of smoking in all public places.	All public places in country are completely smoke-free (or at least 90% of the population covered by complete subnational smoke-free legislation).	Three to seven public places are completely smoke-free, or the law allows designated smoking rooms with strict technical requirements in five or more places.	http://www.who.int/tobacco/global_report/ http://www.who.int/tobacco/global_report/2013/technical_note_i.pdf
5. c	Country has large warnings which are defined as covering on average at least 50% of the front and back of the package with all appropriate characteristics.	Country has large health warnings with all appropriate characteristics; specific health warnings mandated; appearing on individual packages as well as on any outside packaging and labelling used in retail sale; describing specific harmful effects of tobacco use on health; are large, clear, visible and legible (e.g. specific colours and font style and sizes are mandated); whether the warnings rotate; include pictures or pictograms; written in (all) the principal language(s) of the country.	Country has medium-size warnings with some or all appropriate characteristics, or large warnings were missing some appropriate characteristics.	http://www.who.int/tobacco/global_report/ http://www.who.int/tobacco/global_report/2013/technical_note_i.pdf
5.d	Country has a ban on all forms of direct and indirect advertising. Direct advertising bans include: national television and radio; local magazines and newspapers; billboards and outdoor advertising; point of sale. Indirect advertising bans include: free distribution of tobacco products in the mail or through other means; promotional discounts; non-tobacco goods and services identified with tobacco brand names (brand extension); brand names of non-tobacco products used for tobacco products (brand sharing); appearance of tobacco brands (product placement) or tobacco products in television and/or films; and sponsorship, including corporate social responsibility programmes.	Country has a ban on all forms of direct and indirect advertising.	Country has a ban on national TV, radio and print media, but not on all other forms of direct and/or indirect advertising.	http://www.who.int/tobacco/global_report/ http://www.who.int/tobacco/global_report/2013/technical_note_i.pdf
**Source : WHO Country Profiles 2015**				
