# Supplementary material for: Tracing Africa’s progress towards implementing the Non-Communicable Diseases Global action plan 2013–2020: a synthesis of WHO country profile reports
Source: BMC Public Health. 2017 Apr 5;17:297. doi: 10.1186/s12889-017-4199-6 (PMC5382491; doi:10.1186/s12889-017-4199-6)
Supplement: Supplementary file 4 — WHO definitions of indicators and criteria. Description of data: WHO definitions of indicators and criteria_6–7. (PDF 284 kb) [file 12889_2017_4199_MOESM4_ESM.pdf]

Additional file 4: WHO definition of indicators and criteria				
Indicator	Definition	Achievement Criteria		
		Fully achieved	Partly achieved	Links to WHO tools
6.a	Country has a licensing system or monopoly on retail sales of beer, wine, spirits.Country has restrictions for on- / off-premise sales of beer, wine, spirits regarding hours, days and locations of sales. Country has legal age limits for being sold and served alcoholic beverages.	Country has a licensing system or monopoly on retail sales of beer, wine and spirits; and restrictions for on- and off-premise sales of beer, wine and spirits regarding hours and locations of sales and restrictions exist for off-premise sales of beer, wine and spirits regarding days of sales; and legal age limits for being sold and served alcoholic beverages are 18 years or above for beer, wine and spirits.	Country has any but not all the three indicators.	http://www.who.int/entity/substance_abuse/activities/survey_alcohol_health_2012.pdf
6.b	Country has regulatory or co-regulatory frameworks for alcohol advertising through different channels (public service/national TV, commercial/private TV, national radio, local radio, print media, billboards, points of sale, cinema, internet, social media). Country has a detection system for infringements on marketing restrictions	Country has restrictions exist on alcohol advertising for beer, wine and spirits through all channels; and detection system exists for infringements on marketing restrictions.	Country has restrictions on at least public service/national TV, national radio and billboards but no detection system exists for infringements.	http://www.who.int/entity/substance_abuse/activities/survey_alcohol_health_2012.pdf
6.c	Country has excise tax on beer, wine, spirits and adjusts level of taxation for inflation for alcoholic beverages	Country has implemented excise tax on all alcoholic beverages (beer, wine and spirits) is implemented; and adjusted level of taxation for inflation for beer,wine and spirits	Country has excise tax on all alcoholic beverages and adjusted the level of taxation for inflation which is implemented on at least one of the alcoholic beverages	http://www.who.int/entity/substance_abuse/activities/survey_alcohol_health_2012.pdf
7.a	Country has implemented a policy(ies) to reduce population salt/sodium consumption such as product reformulation by industry, regulation of salt content of food, or public awareness programmes.	Country is implementing any policies to reduce population salt consumption	Not defined	http://www.who.int/chp/ncd_capacity/en/
7. b	Country has implemented a policy(ies) to limit saturated fatty acids and virtually eliminate industrially produced trans-fats in the food supply	Country is implementing national policies that limit saturated fatty acids and virtually eliminate industrially produced trans-fats (i.e. partially hydrogenated vegetable oils) in the food supply.	Not defined	http://www.who.int/chp/ncd_capacity/en/
Source : WHO Country Profiles 2015				
