# Supplementary material for: Tracing Africa’s progress towards implementing the Non-Communicable Diseases Global action plan 2013–2020: a synthesis of WHO country profile reports
Source: BMC Public Health. 2017 Apr 5;17:297. doi: 10.1186/s12889-017-4199-6 (PMC5382491; doi:10.1186/s12889-017-4199-6)
Supplement: Supplementary file 5 — WHO definitions of indicators and criteria. Description of data: WHO definitions of indicators and criteria_7c-10. (PDF 287 kb) [file 12889_2017_4199_MOESM5_ESM.pdf]

Additional file 5: WHO definition of indicators and criteria				
Indicator	Definition	Achievement Criteria		
		Fully achieved	Partly achieved	Links to WHO tools
7.c	Country has implemented a policy(ies) to reduce the impact on children of marketing of foods and non-alcoholic beverages high in saturated fats, trans-fatty acids, free sugars or salt.	Country is implementing policies to reduce the impact on children of marketing of foods and non-alcoholic beverages high in saturated fats, trans-fatty acids, free sugars or salt.	Not defined	http://www.who.int/chp/ncd_capacity/en/
7.d.	Country has implemented legislation/regulations that fully implement the International Code of Marketing of Breast-milk Substitutes.	Country implementing the International Code of Marketing of Breast-Milk Substitutes through adoption of national laws	Not defined	http://www.who.int/chp/ncd_capacity/en/
8.	Country has implemented at least one recent national public awareness programme on diet, physical activity or both.	Country has implemented a national public awareness programme on diet within the past 5 years or implemented a national public awareness programme on physical activity within the past 5 years	Not defined	http://www.who.int/chp/ncd_capacity/en/
9.	Government approved evidence-based national guidelines/ protocols/ standards for the management (diagnosis and treatment) of the four main NCDs – cardiovascular disease, diabetes, cancer and chronic respiratory diseases.	Country has national guidelines/protocols/standards exist for all four NCDs (cardiovascular disease, diabetes, cancer and chronic respiratory diseases) which are being partially or fully implemented	Country has guidelines/protocols/standards for at least two of the four NCDs (cardiovascular disease, diabetes, cancer and chronic respiratory diseases), but not for all four, and that these are being partially or fully implemented.	http://www.who.int/chp/ncd_capacity/en/
10.	Country has provision of drug therapy including glycaemic control, and counselling for eligible persons at high risk to prevent heart attacks and strokes, with emphasis on the primary care level.	Country reports that more than 50% of primary health care facilities are offering cardiovascular risk stratification for the management of patients at high risk for heart attack and stroke and that all drugs listed above were generally available in the primary care facilities of the public health sector.	Country reports that between 25% to 50% of primary health care facilities are offering cardiovascular risk stratification for the management of patients at high risk for heart attack and stroke and that all of the drugs listed above were generally available in the primary care facilities of the public health sector	http://www.who.int/chp/ncd_capacity/en/
Source : WHO Country Profiles 2015				
